# Is Cadmium Exposure Associated with the Burden, Vulnerability and Rupture of Human Atherosclerotic Plaques?

**DOI:** 10.1371/journal.pone.0121240

**Published:** 2015-03-27

**Authors:** Göran Bergström, Björn Fagerberg, Gerd Sallsten, Thomas Lundh, Lars Barregard

**Affiliations:** 1 Wallenberg Laboratory, Department of Molecular and Clinical Medicine, Institute of Medicine, Sahlgrenska University Hospital and University of Gothenburg, Gothenburg, Sweden; 2 Occupational and Environmental Medicine, Sahlgrenska University Hospital and University of Gothenburg, Gothenburg, Sweden; 3 Department of Occupational and Environmental Medicine, Lund University Hospital, Lund, Sweden; Stony Brook University, Graduate Program in Public Health, UNITED STATES

## Abstract

The general population is exposed to cadmium from food and smoking. Cadmium is a widely spread toxic pollutant that seems to be associated with cardiovascular diseases, although little is known if it contributes to the occurrence of atherosclerotic plaques and the process whereby plaques become vulnerable and are prone to rupture. We tested the hypotheses that cadmium exposure is associated not only with an increased subclinical burden of atherosclerotic plaques in different vascular territories and early signs of plaque vulnerability, but also with cadmium content and plaque-rupture in the clinical phase of the disease. Ultrasound technique was used to measure plaque prevalence and echogenicity in the carotid and femoral arteries in a population sample of women (n = 599) in whom blood cadmium was measured. In addition cadmium was measured in snap-frozen endarterectomies and whole blood obtained from patients who were referred to surgery because of symptomatic carotid plaques (n = 37). Sixteen endarterectomies were divided into three parts corresponding to different flow conditions and plaque vulnerability. In the population sample blood cadmium was associated with the number of vascular territories with plaques (p = 0.003 after adjustment for potential confounders). The cadmium concentrations in symptomatic plaques were 50-fold higher in plaque tissue than in blood. Cadmium levels in blood and plaque correlated, also after adjustment for smoking and other cardiovascular risk factors (p<0.001). Compared with the other parts of the plaque, the cadmium content was double as high in the part where plaque rupture usually occurs. In conclusion, the results show that cadmium exposure is associated with the burden of subclinical atherosclerosis in middle-aged women with different degrees of glucose tolerance, and that the content of cadmium in symptomatic plaques in patients is related to that in blood, but much higher, and preferentially located in the part of plaque where rupture often occurs.

## Introduction

The general population is exposed to cadmium from food and smoking. The major dietary sources of cadmium are rice, cereals, potatoes, and other root vegetables due to uptake of cadmium from the soil, which is contaminated with fertilizers with high cadmium content and fall-out from industrial emissions and other urban sources [1].

Smoking is a major source of exposure as cadmium in tobacco smoke is effectively absorbed in the lungs [1]. Cadmium exposure is associated with bone damage and fractures, renal damage and certain types of cancer [2]. Accumulating data also indicate that cadmium has pro-atherogenic effects [2–8]. The cadmium contents in urine and blood mirror long term cadmium exposure [1]. Cross-sectional and prospective epidemiological studies using these measures have reported that cadmium exposure levels found in considerable proportions of the population are associated with prevalent and incident cardiovascular mortality and morbidity, independently of smoking and cardiovascular risk factors [2–8]. In an ultrasound study of a female cohort we observed that cadmium exposure was associated with the prevalence and future growth of atherosclerotic plaques in the carotid arteries [9]. Experimental studies in rabbits and mice have demonstrated how cadmium intake enhances the atherosclerotic process [10–12]. The underlying mechanisms have not been clarified, but disruption of the endothelium, increased apoptosis, and oxidative stress promoting inflammation have been reported [10, 13].

The prevalence of atherosclerotic plaques is closely associated with old age and most plaques never cause clinical disease [14]. A key process in the transition to a symptomatic plaque is the occurrence of plaque vulnerability, leading to plaque rupture and thrombosis, which, in the end can cause end-organ diseases such as myocardial infarction or stroke [15]. A thin fibrous cap, neovascularisation, a large lipid core and inflammation are important features of this plaque phenotype [15].

B-mode ultrasound images of carotid atherosclerotic plaques with low echogenicity are associated with histological characteristics typical of vulnerable plaques and cerebrovascular clinical events [16–18]. Such findings, mainly made in clinical studies indicate that low plaque echogenicity can be used as a measure of plaque-vulnerability. Plaques in the femoral artery with low echogenicity are also associated with clinical events and cardiovascular risk factors [19].

Another approach to explore symptomatic atherosclerotic lesions is to study endarterectomies of symptomatic carotid plaques. We, as well as others have previously shown that carotid plaques most often rupture in the upstream part, which is before the blood flow passes the maximum stenosis of the plaque [20–23]. Although a few previous autopsy studies have reported considerable cadmium content in aorta walls [24–27], there is no available report in the literature on cadmium concentrations in atherosclerotic plaques.

In order to better understand how cadmium may cause symptomatic atherosclerosis we hypothesized that cadmium exposure is associated not only with an increased subclinical burden of atherosclerotic plaques in different vascular territories and early signs of plaque vulnerability, but also with cadmium content and rupture of plaques in the late clinical phase. Accordingly our first aim was to examine if cadmium exposure is positively associated with the sub-clinical plaque-burden in the carotid and femoral arteries and the prevalence of low echogenicity plaques. The second, clinical aim was to examine if human symptomatic atherosclerotic plaques have higher concentration of cadmium than in blood, if cadmium levels in blood and carotid plaques correlate, and if the cadmium concentrations within symptomatic plaques are highest in the upstream part were plaque rupture most often takes place. In the present study we succeeded to test these hypotheses in a population-sample of women, and in a biobank of endarterectomies, representing patients who were operated on for symptomatic carotid atherosclerosis.

## Material and Methods

### Participants

The subclinical prevalence of plaque burden and echogenicity were examined in a previously described population-based sample [9, 28, 29]. Briefly, all 64-year old women in Gothenburg, Sweden were in 2001–2003 invited to a screening examination with oral glucose tolerance test. The women with diabetes and similarly-sized randomly selected groups of women with impaired and normal glucose tolerance test were recruited to the study. In 599 out of 629 women it was possible to determine blood cadmium. The baseline examinations included questionnaires concerning health, life style and medication, anthropometrics, blood pressure, venous blood samples for assessment of cardiovascular risk factors and aliquots of whole blood, serum and plasma which were kept frozen at -80°C [9, 28]. Ultrasound examination of both carotid arteries and the right femoral artery was performed for assessment of plaque occurrence and plaque echogenicity [9, 29].

In the clinical substudy of symptomatic carotid plaques we used endarterectomies from the Göteborg Atheroma Study Group (GASG) biobank at the Sahlgrenska University Hospital (Göteborg, Sweden) [23, 30]. This biobank contains blood and endarterectomies that were obtained from patients with symptomatic carotid atherosclerotic disease with high-grade carotid stenosis [23]. The endarterectomies in the present study (n = 37) were snap-frozen in liquid nitrogen immediately after surgical removal and were after that stored in -80°C. Clinical data were obtained through patient questionnaires and medical records. Blood samples were drawn for assessment of cardiovascular risk factors as previously described [30]. Whole blood for measurement of cadmium concentration was available in 35 patients.

### Ethics statement

Both studies have been carried out in accordance with the Declaration of Helsinki and were approved by the Regional Ethical Review Board in Gothenburg and all participants gave written informed consent to participate.

### Cardiovascular risk factors

Smoking history included current, previous and never smoking. Pack years were calculated [9]. Supine blood pressure was measured after supine rest [9]. Fasting blood glucose, apolipoprotein A-I, apolipoprotein B were determined by standardized methods as previously described [28, 30]. Diabetes was diagnosed according to the WHO definition [31].

### Ultrasound imaging

The ultrasound examination of the carotid and femoral arteries was performed with an ultrasound scanner equipped with a linear 8L5-MHz transducer (Sequoia 512, Siemens, Mountain View, California). The examination of the carotid arteries for occurrence of plaques in the near and far wall was performed in accordance with previously published semi-automated methods and definitions [19, 32–34]. A semi-automated method was used for the measurement of the gray-scale median (GSM) of each plaque in the carotid arteries [34]. The upper range of the first tertile of GSM was used as a measure of low plaque echogenicity as previously described (GSM < 42) [34]. The echogenicity of plaques in the femoral arteries was visually classified according to the Grey-Weale method defining class I and II plaques as having low echogenicity [35].

### Preparation of endarterectomies

**Fig 1:**
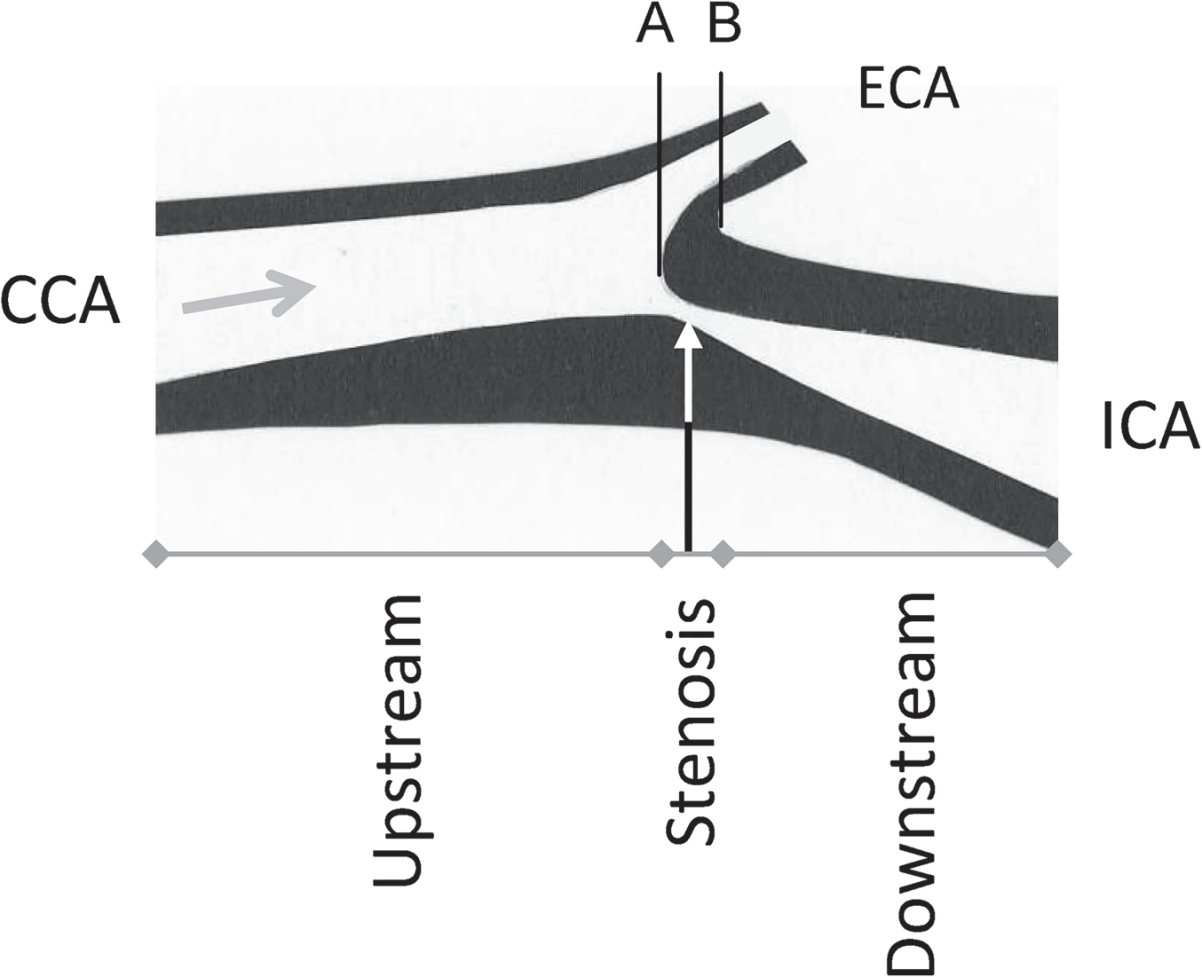
Description of a highly stenotic carotid plaque and the division of the endarerectomies. The figure shows an endarterectomy (an atherosclerotic plaques, excised at operation) and the arrow shows the direction of the blood flow. The endarterectomy samples were prepared in relation to the blood flow direction and the bifurcation between the internal (ICA) and external (ECA) carotid artery. The inner flow divider was defined as the zero point in the vascular lumen, separating the blood flow to ICA and ECA (A) and was localized at the bifurcation (B) minus 1.5 mm [23]. From that zero point the stenosis part of the plaque was defined as -1.5 to 4.5 mm. The upstream part was < -1.5 mm and the downstream part as >4.5 mm of the endarterectomy. CCA—common carotid artery.

The sample consisted of 37 frozen endarterectomies. Sixteen of those allowed full identification of the common, external and internal carotid artery. From a previous study based on MR angiography imaging and serial section of carotid endarterectomy we know that in two thirds of all cases, the maximum stenosis is located 1.5 mm below to 4.5 mm above the flow divider between the external and internal carotid artery [23]. As shown in Fig. 1, this information was used to divide the endarterectomies into three parts: upstream, stenosis, and downstream. The cadmium concentration was examined in each of these parts.

In the remaining 21 carotid endarterectomies it was not technically possible to remove the plaques with a conserved recognizable anatomical structure and cadmium concentration was therefore measured in each endarterectomy without knowledge of which vascular section the plaque originated from.

### Measurements of cadmium

The dry weight of all plaque samples was determined after drying at 90°C for 24 h in a thermostat-controlled cupboard. When the samples had dried, 0.10 ml deionised water was added followed by addition of 0.10 ml with a mixture of concentrated perchloric acid and nitric acid (5:1). The samples were then digested at 110°C for 24 h and thereafter diluted to a volume of 5 ml with deionised water. The blood samples were diluted ten times with an alkaline solution according to Barany et al. [36]. The concentrations of cadmium in plaque and blood samples were determined by inductively coupled plasma mass spectrometry (ICP-MS; Thermo X7, Thermo Elemental, Winsford, UK). The detection limits were 0.050 ng for plaque analysis and 0.05 μg/L for blood analysis. The analytical accuracy was checked against reference materials. For plaque analysis freeze-dried muscle tissue (Community of Reference, Brussels, BCR 184) were used (n = 2) and the results obtained were 12.2 and 10.7 vs. certified 13±2 ng cadmium/g.

Two reference materials were used for the blood analysis. For Seronorm Trace Elements Whole Blood L-1 (SERO AS, Billingstad, Norway), lot. 1103128, the result (μg cadmium/L, mean±SD) obtained was 0.34±0.02 (n = 4) vs. recommended 0.32–0.40, and for Human Blood Reference Samples, lot. C-06-16 (Centre de Toxicologie du Quebec, International Comparison Program, Canada) the result was 1.06±0.02 (n = 4) vs. recommended 1.1±0.13. All blood samples were prepared in duplicate and the method imprecision (calculated as the coefficient of variation for duplicate preparations) was 3.1%

Cadmium in whole blood in the 599 women was analysed with ICP-MS as described above, for further details see [9].

### Statistical methods

Statistical analyses were performed with SPSS18.0 (SPSS Inc., Chicago, IL). Results are presented as number (%), means (standard deviation), or geometric mean (standard error) for skewed data. Blood cadmium concentrations are presented as geometric mean (5 and 95 percentile). Chi-square, ANOVA and trend-tests were used to compare characteristics of participants by cadmium tertiles. Student’s t-test was used for comparison of continuous variables. Pearson´s correlation coefficients were calculated and linear multiple regression with log-transformation of skewed variables and logistic regression for calculation of odds ratio (95% confidence interval) were used. The variables introduced in the statistical model were risk factors for cardiovascular disease. Two-tailed p<0.05 was considered statistically significant.

## Results

### Subclinical plaques: blood cadmium, plaque burden and echogenicity

The geometric mean of the blood cadmium concentration was 0.38 (5–95 percentiles 0.14–1.69) μg/L. As shown in Table 1, apart from smoking, no other cardiovascular risk factor was significantly associated with blood cadmium. Increasing levels of blood cadmium concentrations were in univariate analysis associated with an increased number of vascular territories with atherosclerotic plaques. There was also an association between cadmium concentrations and the prevalence of low echogenicity femoral plaques.

**Table 1:** Characteristics of participants in the substudy of subclinical plaques in a population-based cohort.

	**Tertiles of blood cadmium**			**p for trend**
	**1 (n = 200)**	**2 (n = 199)**	**3 (n = 200)**	
Blood cadmium, μg/L (geometric mean, 5–95 percentiles)	0.18 (0.12–0.25)	0.34 (0.26–0.44)	0.91 (0.47–2.48)	-
Never smoking history, n (%)	141 (71)	101 (51)	25 (13)	0.005^a^
Previous smoking history, n (%)	58 (29)	90 (45)	61 (31)	
Current smoking history, n (%)	1 (1)	8 (4)	114 (57)	
Pack years	36 (91)	125 (95)	454 (359)	0.005
Waist, cm	94 (13)	93 (92)	93 (12)	n.s.
Systolic blood pressure, mm Hg	146 (19)	145 (18)	143 (18)	n.s.
Serum apolipoprotein B/apolipoprotein A-1	0.75 (0.21)	0.75 (0.23)	0.75 (0.21)	n.s.
Diabetes, n (%)	81 (41)	63 (32)	74 (37)	n.s.
Statin treatment, n (%)	25 (13)	25 (13)	22 (11)	n.s.
Number of vascular territories^b^ with prevalent plaques, n (%)	106 (53)	91 (46)	56 (28)	0.005^c^
One vascular territory with prevalent plaques, n (%)	77 (39)	81 (41)	92 (46)	
Two vascular territories with prevalent plaques, n (%)	17 (9)	27 (14)	52 (26)	
Prevalence of low echogenicity carotid plaques, n (%)	23 (38)	18 (25)	31 (34)	n.s.
Prevalence of low echogenicity femoral plaques, n (%)	27 (60)	33 (69)	61 (77)	0.042

^a^ p-value (Chi-square) refers to never, previous, current smoking

^b^ The vascular territories are the left or right carotid arteries and the right femoral artery and the alternatives are no, one and two territories with prevalent plaques.

^c^ p-value (Chi-square) refers to numbers (%) of vascular territories with no, one and two prevalent plaques

Characteristics, burden of atherosclerotic plaques and signs of plaque vulnerability in the carotid and right femoral artery territories among 64-year old women by tertiles of blood cadmium concentrations. Values are mean (standard deviation) unless else is indicated.

A linear regression analysis was performed with the number of arterial segments having plaques in each participant as dependent variable and log blood cadmium, smoking history, pack years, log apolipoprotein B/lipoprotein A-I, systolic blood pressure, diabetes, log waist and statin treatment as independent variables. The results showed that log blood cadmium was associated with number of arterial territories with plaque (part r = 0.12, p = 0.003; beta-coefficient 0.39, 95% confidence interval 0.13 to 0.64). In the subgroup of never-smokers the corresponding beta-coefficient was 0.09 (95% confidence interval -0.34 to 0.52). In a logistic regression analysis the odds ratio (OR) for low echogenicity plaques in the carotid and femoral arteries were 1.2 (95% CI 0.4–3.4) and 0.9 (95% CI 0.3–2.9), respectively, when tertiles 3 and 1 of blood cadmium were compared and adjustment was made for the same independent variables as in the linear regression.

### Clinical plaques: cadmium in blood and carotid plaques

The geometric mean of the blood cadmium concentration was 0.45 (5–95 percentiles 0.16 to 1.67) μg/L. The cadmium concentrations in all plaques varied from 0.0072 to 0.0819 μg/g wet weight (geometric mean 0.022 [5–95 percentiles 0.008–0.074]) and from 0.010 to 0.29 μg/g dry weight (geometric mean 0.069 [5–95 percentiles 0.019–0.226]).

The characteristics of the patients are shown in Table 2. The clinical diagnoses caused by the symptomatic carotid plaques were transient ischemic attacks (49%), amaurosis fugax (30%), minor stroke (19%), and unclassified (3%).

**Table 2:** Characteristics of the patients with symptomatic carotid plaques in the clinical substudy (n = 37).

**Characteristic**	**Mean (SD) or n (%)**
Female sex, n (%)	12 (32.4)
Age, years, (range, SD)	70.1 (51–85, 8.2)
Never smoking history, n (%)	5 (13.5)
Previous smoking history, n (%)	18 (48.6)
Current smoking history, n (%)	13 (35.1)
Pack years of smoking, median (range)	27 (0–90)
Hypertension, n (%)	29 (78.4)
Diabetes, n (%)	6 (16.2)
Previous myocardial infarction, n (%)	10 (27.0)
Statin treatment, n (%)	27 (73.0)
Apolipoprotein B/A-I ratio	0.77 (0.20)

**Fig 2:**
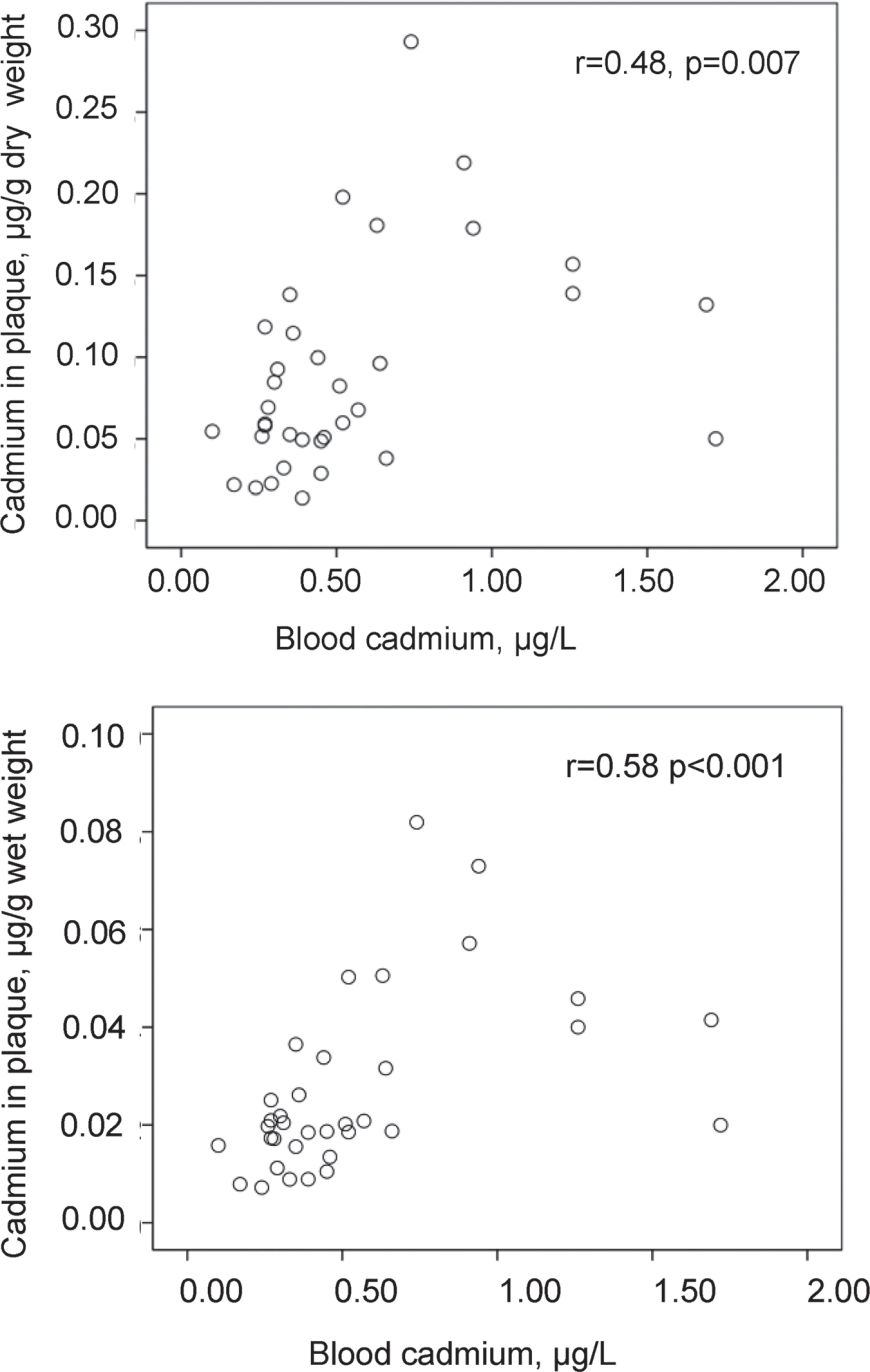
Correlation between cadmium levels in blood and atherosclerotic plaques (endarterectomies). Scatterplot of cadmium concentrations in blood in relation to those in carotid endarterectomies by g of dry and wet weight (n = 35).

Blood concentrations of cadmium correlated with the cadmium content in the plaques, both by wet and dry weight (Fig. 2). Smoking history (never, previous, and current smoking) correlated to blood cadmium (p<0.001), and with borderline significance to plaque cadmium/wet weight (p = 0.052). Pack years of smoking were associated with blood cadmium with borderline significance (r = 0.35, p = 0.060), and with plaque cadmium/wet weight (r = 0.58, p = 0.035). The apolipoprotein B/A-I ratio showed the following correlations: with blood cadmium (r = 0.17, n.s.), with plaque cadmium/wet weight (r = 0.35, p = 0.035). The concentrations of cadmium in blood or plaques showed no further associations with the patient characteristics that are presented in Table 1 (data on associations not shown).

In a multiple linear regression analysis, log cadmium in plaques/wet weight was associated with log blood cadmium (partial correlation coefficient = 0.56, p<0.001) after adjustment for smoking history and apolipoprotein B/A-I ratio (R^2^ = 0.46). This partial correlation coefficient was 0.52 (p<0.001) after further adjustment for sex, age, statin treatment, prevalent hypertension or diabetes (R^2^ = 0.60). Similar results were obtained for cadmium content in plaque by dry weight and when smoking history was replaced by pack years of smoking (data not shown).

### Clinical plaques: cadmium distribution

**Fig 3:**
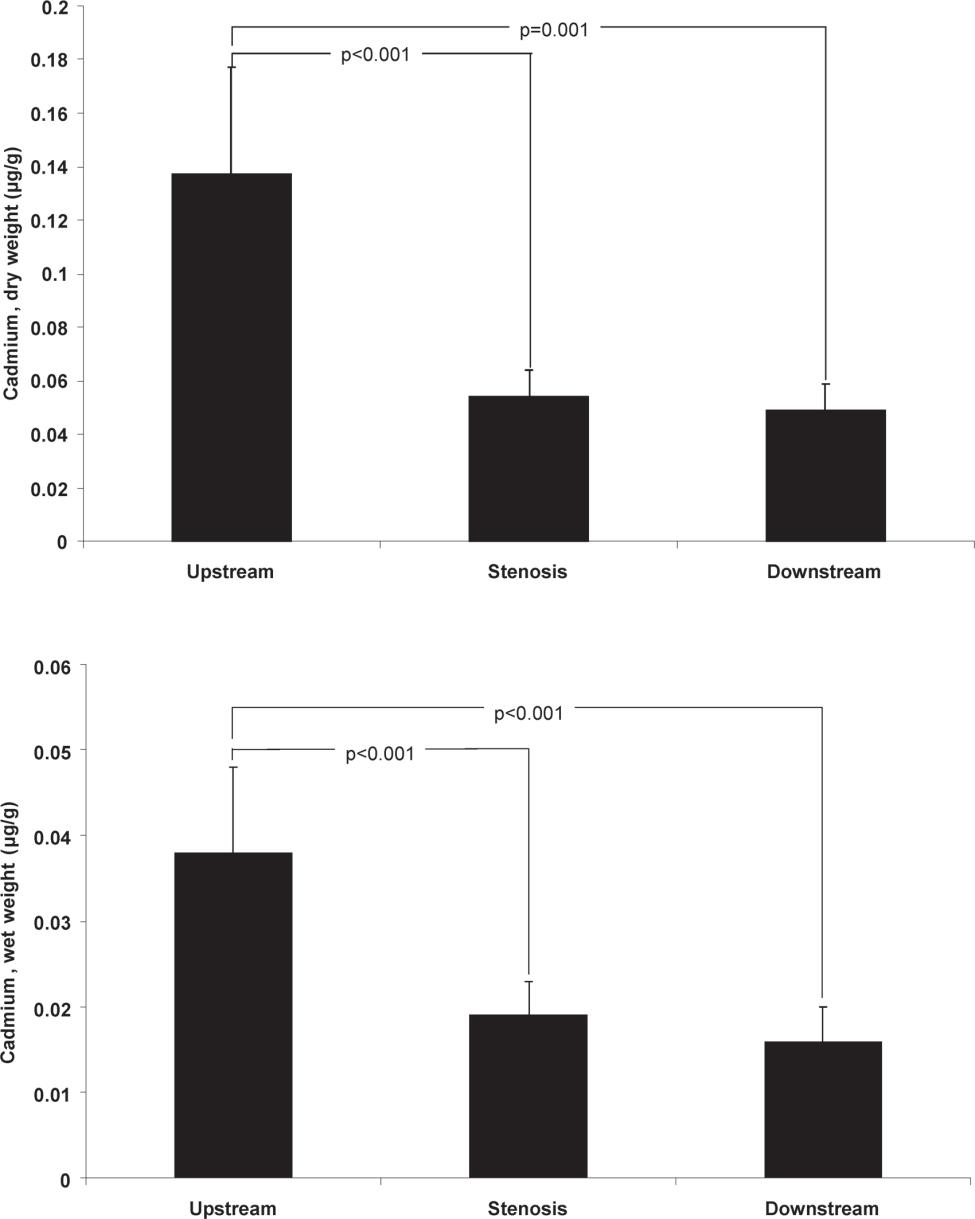
Concentrations of cadmium in different sections of symptomatic carotid plaques. The concentrations of cadmium by g of dry and wet weight in the upstream, stenosis, and downstream sections of endarterectomies from patients with symptomatic carotid stenosis (n = 16). Data are geometric mean (SE).

The distribution of cadmium is shown in Fig. 3. The concentration of cadmium was higher in the upstream section than in the stenosis or downstream sections. In 15 of 16 patients’ cadmium levels by wet weight were higher in the upstream than in the stenosis section (93.8%), and in 13 of 16 patients compared with the downstream section (81.3%). The corresponding proportions were similar for cadmium levels by dry weight (data not shown).

### Clinical plaques: never smoking group

In the group who had never smoked (n = 5) the geometric mean (SE) of the cadmium concentration/g wet plaque weight was 0.014 (0.002) μg/g, to be compared with 0.022 (0.004) and 0.027 (0.006) μg/g in the previous (n = 18) and current (n = 13) smokers. The corresponding data for cadmium levels by dry weight were 0.045 (0.014), 0.071 (0.016), and 0.082 (0.019) μg/g, respectively. Similarly, the geometric means of blood cadmium levels were 0.26, 0.37 and 0.76 μg/L in the never, previous, and current smoking groups. Hence, in never-smokers there was a 50-fold increase in the concentration of cadmium in plaque tissue, compared with the blood level.

In one never-smoking patient it was possible to examine the concentrations of cadmium within a carotid plaque. The distribution was exactly as in the total group with a high cadmium level in the upstream section (0.034 μg/g wet weight) and low levels in the stenosis and downstream sections (0.008 and 0.007 μg/g wet weight, respectively).

## Discussion

The results of the present study showed that increasing levels of blood cadmium were associated with increasing numbers of arterial territories with subclinical atherosclerotic plaques in the carotid and femoral arteries. This finding extends our previous observations of an association between cadmium exposure and carotid plaques and supports the concept of a pro-atherogenic effect [9, 29, 37]. We also investigated if cadmium exposure could be related to indices of plaque vulnerability in two different models. While we failed to show an association between cadmium exposure and low echogenicity in small to moderate-sized subclinical carotid plaques after adjustment for potential confounders, our findings in the clinical substudy suggested a potential role in vulnerability Thus, in patients who underwent carotid endarterectomy because of symptomatic atherosclerotic lesions the results showed that the content of cadmium within the plaque was highest in the upstream part, where plaques most often rupture and cause clinical disease. The failure to show a relationship between cadmium exposure and low echogenicity has a plausible explanation in the fact that we examined small plaques whereas the documented associations between low echogenicity, histological features typical of plaque vulnerability and clinical events are based upon studies of patients with larger and more advanced stenotic plaques [16–18].

There are autopsy studies from Finland, the Netherlands and England in which cadmium concentrations were measured in different organs or in the aorta [24–27]. Taken together, the mean cadmium level in abdominal aorta in those studies ranged from 0.16 to 0.36 μg/g (wet weight). In our study the arithmetic mean of the cadmium levels in carotid plaques was 0.027 μg/g (wet weight). However, these cadmium concentrations are not comparable. Firstly, we measured cadmium levels in the atherosclerotic plaque which is a pathological tissue located in the intima-layer, whereas the mentioned studies examined whole arteries encompassing all [24–27] or separate [27] layers of the vascular wall. Secondly, the cadmium concentration in the aorta may not be representative of cadmium in the carotid artery. An experimental study of dogs in which cadmium was administered intra-peritoneally indicated that there may be differences in arterial cadmium content between arteries of larger and smaller size [38]. We observed that our symptomatic carotid plaques have a cadmium concentration about 50 times higher than that in circulating blood (0.022 μg/g vs. 0.45 μg/L). Moreover, these cadmium levels in plaque correlate also with the concentrations of cadmium in blood. These findings support the concept that cadmium is accumulated in plaque tissue and may reach critical concentrations, having direct effects on the atherosclerotic process.

We interpret our results as supporting the hypotheses that cadmium may have both pro-atherogenic effects and effects on the mechanisms leading to plaque rupture. Firstly, our results are in line with previous data showing that cadmium exposure enhances the atherosclerotic process in experimental models [10–12] and is associated with increased prevalence of intermittent claudication, that is atherosclerotic disease in leg arteries [4, 29], and asymptomatic carotid artery disease [9, 37]. Secondly, as shown in a recent meta-analysis cadmium exposure is associated with the morbidity and mortality in coronary heart disease, in which plaque vulnerability and plaque rupture are key factors [39]. Plaque rupture is similarly important in ischemic stroke caused by symptomatic atherosclerotic disease in the carotid artery [15]. We have previously shown that in symptomatic carotid plaques, the upstream part of the plaques have the highest content of intra-plaque haemorrhage and surface thrombosis [23]. This will result in an accumulation of erythrocytes which contain cadmium, but this cannot explain our findings. Hence, the mechanism underlying the accumulation of cadmium in the particular section of the plaque where rupture occurs is not known.

For initiation of atherosclerosis the passage of proatherogenic lipoproteins and monocytes through the endothelial barrier and retention in the intimal layer seem to be key mechanisms which lead to an inflammatory response and development of plaques [40]. In support of this concept cadmium exposure has been found to cause endothelial damage and increased permeability in vivo and in vitro and to enhance atherosclerotic plaques in coronary arteries in rabbits and in the aorta of ApoE–/—mice [10, 11, 41]. Increased oxidative stress after cadmium administration has been demonstrated in rats [12]. Cadmium seems to disrupt cadherin-dependent endothelial cell junctions [10]. Uptake of cadmium in endothelial cells causes DNA-damages and atypical apoptosis and enhances the endothelial cell expression of the vascular cell adhesion molecule 1 (VCAM-1) thereby facilitating adhesion and migration of immune cells into the vessel wall [10–11].

It is a limitation that this is a cross-sectional study and it is not possible to clarify causal mechanisms. Moreover, it is important to consider the confounding effect of smoking, which is a major source of cadmium exposure together with cadmium intake by food. However, previous large prospective epidemiological studies have shown that cadmium exposure is associated with future cardiovascular disease also in never-smokers, particularly in men [3–8]. In the present clinical study adjustment for smoking history or other cardiovascular risk factors did not change the association between blood and plaque cadmium concentrations. A subgroup analysis of patients who had never smoked showed that in this group the levels of cadmium in blood as well as in plaque tissue were lower than corresponding levels in ever-smokers. However, in never-smokers the cadmium levels in plaque tissue were still 50 times higher than those in blood. In addition the cadmium concentration in a never-smoker showed the same distribution within the carotid plaque as in the total group. Taken together, these data indicate that our observations are not explained by smoking *per se*.

In the subclinical study, a subgroup analysis of the never smokers showed that the 95% confidence interval of the beta-coefficient overlapped with that in the total group, indicating that with a larger study it would have been possible to demonstrate if there was an association between blood cadmium and the number of vascular territories with prevalent plaques. The study of subclinical plaques was performed in women and the results cannot be inferred to men, although the pro-atherogenic effects were shown in the sex with the smallest risk for cardiovascular disease.

We conclude that our data extend the knowledge from epidemiological and experimental studies of cadmium as a risk factor for atherosclerotic diseases. We have shown that increasing cadmium exposure is associated with increased burden of subclinical atherosclerotic plaques and that symptomatic carotid plaques contain cadmium in levels much higher than those in blood, that cadmium concentrations in blood and plaques correlate and that within the plaques the cadmium concentration is highest in upstream section, where plaque vulnerability often is most pronounced.
